# Review of Literature of Radial Nerve Injuries Associated with Humeral Fractures—An Integrated Management Strategy

**DOI:** 10.1371/journal.pone.0078576

**Published:** 2013-11-08

**Authors:** YuLin Li, GuangZhi Ning, Qiang Wu, QiuLi Wu, Yan Li, ShiQing Feng

**Affiliations:** Department of Orthopaedics, Tianjin Medical University General Hospital, Tianjin, PR China; University Hospital La Paz, Spain

## Abstract

**Background:**

Radial nerve palsy associated with fractures of the shaft of the humerus is the most common nerve lesion complicating fractures of long bones. However, the management of radial nerve injuries associated with humeral fractures is debatable. There was no consensus between observation and early exploration.

**Methods and Findings:**

The PubMed, Embase, Cochrane Central Register of Controlled Trials, Google Scholar, CINAHL, International Bibliography of the Social Sciences, and Social Sciences Citation Index were searched. Two authors independently searched for relevant studies in any language from 1966 to Jan 2013. Thirty studies with 2952 humeral fractures participants were identified. Thirteen studies favored conservative strategy. No significant difference between early exploration and no exploration groups (OR, 1.03, 95% CI 0.61, 1.72; I^2^ = 0.0%, p = 0.918 n.s.). Three studies recommend early radial nerve exploration in patients with open fractures of humerus with radial nerve injury. Five studies proposed early exploration was performed in high-energy humeral shaft fractures with radial nerve injury.

**Conclusions:**

The conservative strategy was a good choice for patients with low-energy closed fractures of humerus with radial nerve injury. We recommend early radial nerve exploration (within the first 2 weeks) in patients with open fractures or high-energy closed fractures of humerus with radial nerve injury.

## Introduction

Fractures of the shaft of the humerus account for 1% to 3% of all fractures [1]. Early studies suggested that high-energy trauma and injuries in younger patients were more likely associated with this fracture [2]. With acceptable reduction and union, most humeral shaft fractures can be treated conservatively. However, surgical management is required in special situations such as polytrauma, open or bilateral fractures, floating elbow, and obesity. With the development of internal fixation technology, indications for operation expand while new debates on procedure choice appear [3].

Radial nerve palsy associated with fractures of the shaft of the humerus is the most common nerve lesion complicating fractures of long bones [4]. These can be divided into three categories depending on occurrence time: primary, delayed and secondary. However, the optimal management strategy for radial nerve palsy in the setting of a humeral shaft fracture remains controversial. Nerve function recovery is often spontaneous in closed fractures within a period ranging from few weeks to several months [5]. In most cases the radial nerve is intact and the prognosis for complete or functionally useful recovery is favourable. Radial nerve transection is uncommon and is usually associated with an open fracture. Exploration of open fractures with radial nerve dysfunction is now generally agreed on, but all of the recommendations for exploring the radial nerve in closed injuries have been challenged [6].

The purpose of this study was to discuss an integrated management strategy for determining the management procedure when deal with all kinds of humeral fractures with complete sensory and motor radial nerve palsy.

## Methods

### Search Strategy

The PubMed, Embase, Cochrane Central Register of Controlled Trials, Google Scholar, CINAHL, International Bibliography of the Social Sciences, and Social Sciences Citation Index were searched. Two authors independently searched for relevant studies in any language from 1966 to Jan 2013. The search strategy was created with the assistance of a librarian using a combination of terms including “humeral” or “humerus” or “shaft” or “diaphysis” or “fracture” or “radial nerve” or “palsy” or “paralysis”, and “epidemiology”. Additional strategies included hand searches of journals that were not indexed in the electronic sources, internet searches for grey literature, and screening of reference lists of retrieved studies.

#### Selection criteria

Two reviewers independently assessed the titles and abstracts of the publications produced by the initial search strategy. To be eligible for inclusion, studies had to meet the following criteria: (1) describe an original study involving radial nerve injuries associated with humeral fractures (2) report the epidemiological data or the treatment of humeral fractures with radial nerve injuries and (3) retrospective studies or randomised controlled trials. General population studies were eligible for inclusion.

#### Selection of studies

Two reviewers (Li and Ning) independently screened the titles and abstracts of studies identified by the search strategy and discarded clearly irrelevant studies. The same two reviewers also independently applied the selection criteria to the studies retrieved by the literature search. They discussed to resolve any disagreement; if any uncertainty remained, they consulted further reviewer and expert (Feng) to decide.

#### Data extraction and management

Two reviewers independently extracted the data using a standardized form regarding inclusion criteria (study design, participants, essential information, epidemiological data, interventions, and outcomes). A consensus method was used to resolve disagreements, and a third reviewer was consulted if disagreements persisted.

#### Statistical analysis

For dichotomous variables, we derived the relative risks and 95% confidence intervals for each outcome. For continuous variables, we calculated the mean differences and 95% confidence intervals for each outcome. We performed the meta-analysis using a fixed-effect model if no significant heterogeneity was present. To assess heterogeneity between studies, we performed a chi-square test and estimated the I2 statistic. A random effects model was selected to account for heterogeneity in the design and patient selection among included studies. And the subgroup analyses were conducted for different outcomes.

## Results

### Search Results

**Figure 1:**
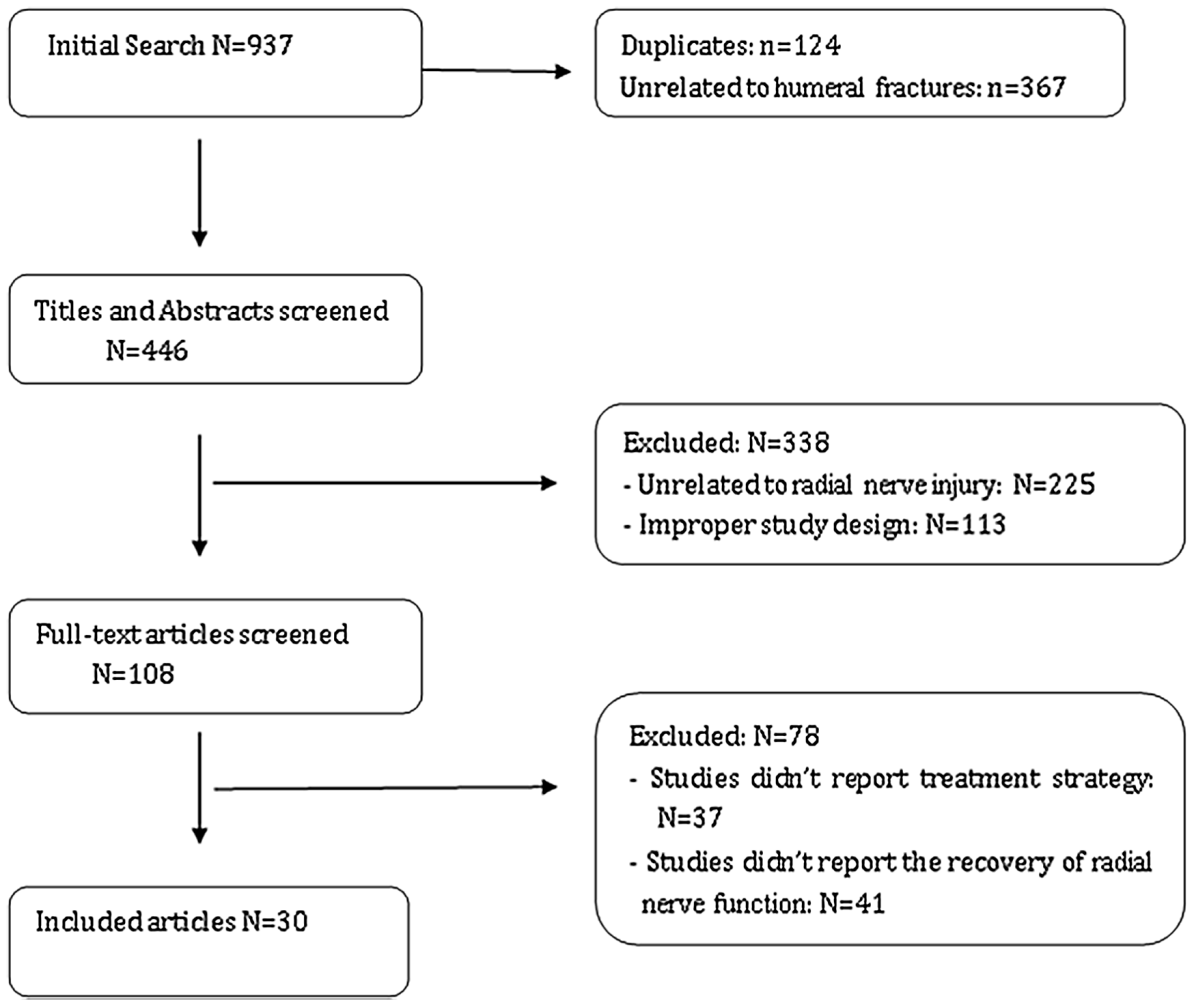
Flowchart of trials selection process.

A search of comprehensive databases retrieved 937 articles. We excluded 124 duplicate articles and 367 unrelated articles. After reviewed the titles and abstracts, 108 articles were included. Then, by reading the whole paper, we included 30 papers. These studies included a total population of 2952 humeral fractures participants. Figure 1 summarizes the study selection process.

### Characteristics of Included Studies

In the thirty studies, 2952 humeral fractures participants were identified. Twenty-six studies were published in English [7]–[16], [18]–[20], [22], [23], [26]–[36] and four studies [17], [21], [24], [25] were published in German. All were retrospective studies. After comparing these articles, all sorts of controversies were appeared. Thirteen [7]–[13], [15], [16], [18], [19], [26], [27] favored a conservative strategy; delayed exploration was performed if no nerve recovery was found with 5–8 weeks. Three [14], [22], [23] recommend early radial nerve exploration (within the first 2 weeks) in patients with open fractures of humerus with radial nerve injury. Five [28], [31]–[34] proposed early exploration was performed in high-energy humeral shaft fractures. One discussed an observation and delayed exploration strategy after secondary radial nerve palsy. And eight [17], [20], [21], [24], [25], [29], [30], [35], [36] did not state a clear preference.

### Epidemiology

The epidemiological data was shown in Table 1. In thirty studies, Seventeen reported the average age was 38.32. A sex ratio of M:F 1.67∶1 was shown in twenty studies, which is composed by 1070 male and 639 female. Left humerus was involved in 518 patients as well as right in 568 patients (L: R 1∶1.1). The ratio in open (309 patients) and closed (927 patients) fracture was 1∶3. Fifteen studies reported total 207 patients involved the proximal third of the humerus, 683 in middle third and 432 in distal third (P:M;D 1: 3.3: 2.1). The mechanism of injury was reported in 14 studies: traffic accident for 436 patients, fall down for 306 patients, crush injury for 30 patients, gunshot injury for 142 patients and 153 patients for other mechanism of injury. Thirteen described a total of 1882 fractures of the shaft of the humerus and 307 radial nerve palsies, giving an overall prevalence of radial nerve palsy of 16.3%.

**Table 1:** Epidemiological data of included studies.

Author	Study design	Included patients	Mean age(year)	Male/Female	Side of fracture		Fracture type		Side of fracture			radial nerve injury		Energy type		Type of injury				
					Left	Right	Open	Closed	Proximal	Middle	Distal	With	Without	High	Low	Traffic	Fall	Crush	Gunshot	Others
Klenerman L [7]	RS	98	–	55/43	–	–	–	–	26	44	29	10	88	–	–	–	–	–	–	–
Kettelkamp DB [8]	RS	216	–	–	–	–	–	–	5	23	5	33	183	5	28	20	9	1	3	0
Shaw JL [9]	RS	45	–	28/17	–	–	8	37	3	42	0	45	0	7	38	13	23	0	0	9
Packer JW [10]	RS	31	52	16/15	–	–	–	–	2	20	9	31	0	–	–	6	21	0	0	4
Mast JW [11]	RS	240	–	–	–	–	67	173	–	–	–	42	198	–	–	–	–	–	–	–
Dameron TB [12]	RS	100	–	–	–	–	–	–	26	51	24	15	85	–	–	–	–	–	–	–
Pollock FH [13]	RS	24	38	13/10	–	–	3	21	2	8	14	24	0	–	–	11	6	0	2	5
Shah JJ [14]	RS	62	–	39/23	33	29	15	47	–	–	–	62	0	–	–	37	9	3	8	5
Böstman O [15]	RS	59	36	45/14	–	–	10	49	1	30	28	59	0	–	–	35	7	17	0	0
Sonneveld GJ [16]	RS	111	43	10/7	6	11	1	16	0	16	1	17	94	–	–	5	2	0	1	9
Rommens PM [17]	RS	78	–	–	–	–	–	–	–	–	–	16	62	–	–	–	–	–	–	–
Samardzić M [18]	RS	91	–	–	–	–	–	–	0	27	10	37	0	–	–	–	–	–	–	–
Sarmiento A [19]	RS	65	28	37/28	22	43	11	54	–	–	–	15	70	–	–	26	18	4	8	9
Bleeker WA [20]	RS	237	35	146/91	–	–	–	–	43	94	31	40	197	58	179	–	–	–	–	–
Kwasny O [21]	RS	35	–	–	–	–	–	–	–	–	–	35	0	–	–	–	–	–	–	–
Amillo S [22]	RS	12	27	10/2	–	–	3	9	3	2	7	12	0	–	–	5	3	0	0	4
Foster RJ [23]	RS	14	29	8/6	–	–	9	5	–	–	–	14	0	–	–	11	0	0	0	3
Marty B [24]	RS	61	–	–	–	–	–	–	–	–	–	9	52	–	–	–	–	–	–	–
Goldhahn S [25]	RS	67	–	–	–	–	–	–	–	–	–	12	55	–	–	–	–	–	–	–
Ogawa K [26]	RS	90	25	89/1	10	80	–	–	–	–	–	14	76	–	–	–	–	–	–	–
Larsen LB [27]	RS	26	21	19/7	–	–	–	–	–	–	–	26	0	–	–	–	–	–	–	–
Sarmiento A [28]	RS	620	36	391/229	317	303	155	465	92	303	219	67	553	–	–	211	192	0	118	99
Kim DH [29]	RS	260	42	–	–	–	–	–	–	–	–	260	0	–	–	–	–	–	–	–
Lin J [30]	RS	21	–	12/9	9	12	–	–	0	2	19	21	0	–	–	–	–	–	–	–
Ring D [31]	RS	24	27	18/6	–	–	11	13	–	–	–	24	0	24	0	16	4	2	2	0
Noaman H [32]	RS	36	30.3	22/14	–	–	7	29	2	11	23	36	0	–	–	25	5	0	0	6
Venouziou AI [33]	RS	18	32.2	15/3	–	–	9	9	–	–	–	18	0	13	5	–	–	–	–	–
Mahabier KC [34]	RS	186	58.7	80/106	106	80	–	–	–	–	–	17	169	32	134	–	–	–	–	–
Korompilias AV [36]	RS	25	36	17/8	15	10	–	–	2	10	13	25	0	20	5	15	7	3	0	0

RS: Retrospective study.

### Conservative Strategy

**Figure 2:**
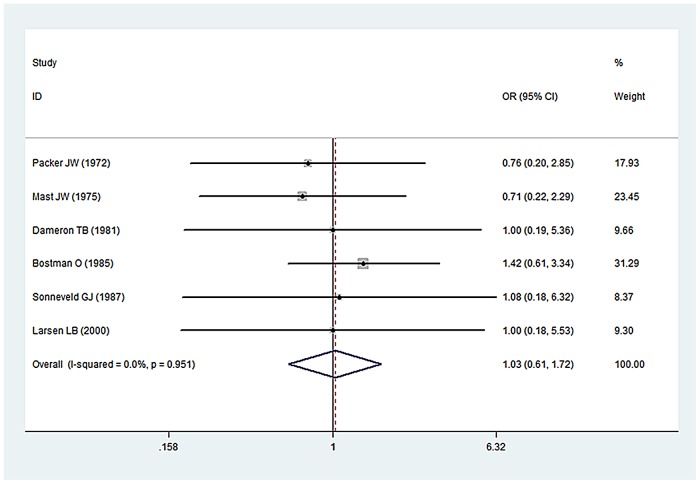
Forest plots of pooling nerve functional recovery between early exploration groups and no exploration groups.

**Figure 3:**
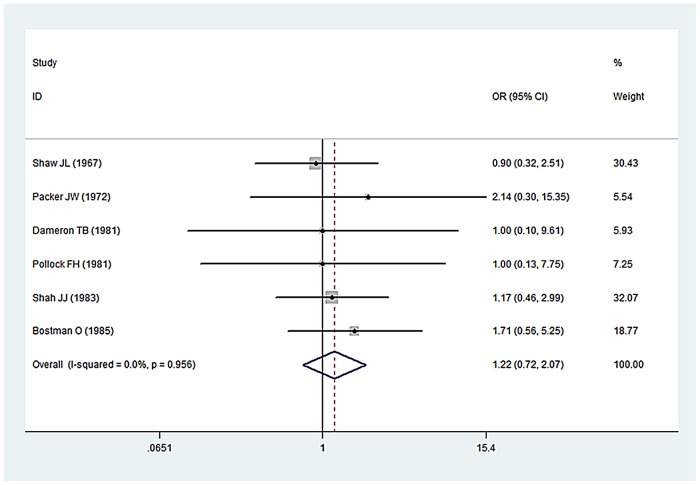
Forest plots of pooling nerve functional recovery between delay exploration groups and no exploration groups.

Thirteen [7]–[13], [15], [16], [18], [19], [26], [27] studies favored conservative strategy; delayed exploration was performed if no nerve recovery was found with 5–8 weeks. In these studies, Six [10]–[12], [15], [16], [27] compared nerve functional recovery of early exploration with no exploration. A pooled analysis of the studies found no significant difference between these two groups (OR, 1.03, 95% CI 0.61, 1.72; I^2^ = 0.0%, p = 0.918 n.s.) (Fig. 2). And Six [9], [10], [12]–[15] compared nerve functional recovery of delay exploration with no exploration. A pooled analysis of the studies found no significant difference between these two groups (OR, 1.22, 95% CI 0.72, 2.07; I^2^ = 0.0%, p = 0.453 n.s.) (Fig. 3). Eleven [8]–[12], [15], [16], [18], [19], [26], [27] studies reported the damage degree of radial nerve after exploration. Total 134 patients with radial nerve injury accepted early or delay exploration. In 15 cases the nerve was totally divided (11.19%), and 119 cases were partially divided or integrated.

### Early Exploration in Open Fractures

Three [14], [22], [23] studies recommend early radial nerve exploration (within the first 2 weeks) in patients with open fractures of humerus with radial nerve injury. Twenty-four patients with open fractures accepted exploration. In 8 cases the nerve was totally divided (33.3%), in 3 cases the nerve found entrapped in fragments, 5 cases were partially divided and 8 cases were integrated.

### Early Exploration in High-energy Fractures

Falls from the standing position was defined as low energy trauma, whereas motor vehicle accidents, falls from height and crushing injuries were considered as high energy trauma. Five [28], [31]–[34] studies proposed early exploration was performed in high-energy humeral shaft fractures. Total 65 patients with high-energy fractures were accepted early exploration. In 25 cases the nerve was totally divided (38.5%), in 4 cases the nerve found entrapped in fragments, 20 cases were partially divided and 16 cases were integrated.

## Discussion

The management of radial nerve injuries associated with humeral fractures is debating. There was no consensus between observation and early exploration. The principal advantage of observation is the fact that most nerves are intact and recover, and the principal advantage of early exploration is that it may provide the best opportunity for nerve recovery when a nerve laceration is identified and grafted in a timely fashion. In this study, we aim to propose an integrated management strategy for this injury.

The diagnosis for radial nerve injury after humeral fractures is easy, however, the verify of damage degree of the radial nerve is very difficult. It is the degree for damage of radial nerve playing a decisive role in the judgment of whether exploration is necessary or not. Bodner G et al reported the feasibility of using ultrasonography (US) for evaluation of a radial nerve injury associated with humeral shaft fracture. In five patients, US findings of a severely damaged radial nerve were confirmed at surgical nerve inspection. In one patient, the nerve was entrapped between fragments. One patient had a complete nerve dissection, one had a lacerated nerve from a loose compression plate, and one had a nerve riding on the edge of a bone fragment. In the fifth patient who underwent surgical inspection, the nerve was buried in the callus. In the six patients treated conservatively, US showed continuity of the nerve. The results suggested that US may be useful for accurate evaluation of the radial nerve in patients with nerve palsy associated with humeral shaft fracture.

After analyzing the thirteen studies [7]–[13], [15], [16], [18], [19], [26], [27] favored conservative strategy, the pooled analysis of the studies found no significant difference of nerve functional recovery between early exploration groups (within 2 weeks) and no exploration groups. And the same results were proven between delay exploration groups (over 2 weeks) and no exploration groups. Most authors recommended initial observation was the preferred strategy, because of the high intact and recover rate of radial nerve. However in three studies [14], [22], [23], the early exploration in patients with open fractures of humerus with radial nerve injury was commendatory. Because in 24 patients with open fractures accepted exploration, 8 cases were totally divided (33.3%) and 3 cases the nerve found entrapped in fragments. All these 11 patients (45.8%) need surgical repair or reconstruction of the nerves; it was far higher than the rate in conservative groups (11.19%). In other five studies [28], [31]–[34], early exploration in high-energy humeral shaft fractures was performed. Total 65 patients with high-energy fractures received early exploration. The nerve was totally divided in 25 cases (38.5%), in 4 cases the nerve found entrapped in fragments. 29 of the 65 patients (44.6%) need surgical repair or reconstruction of the nerves.

**Figure 4:**
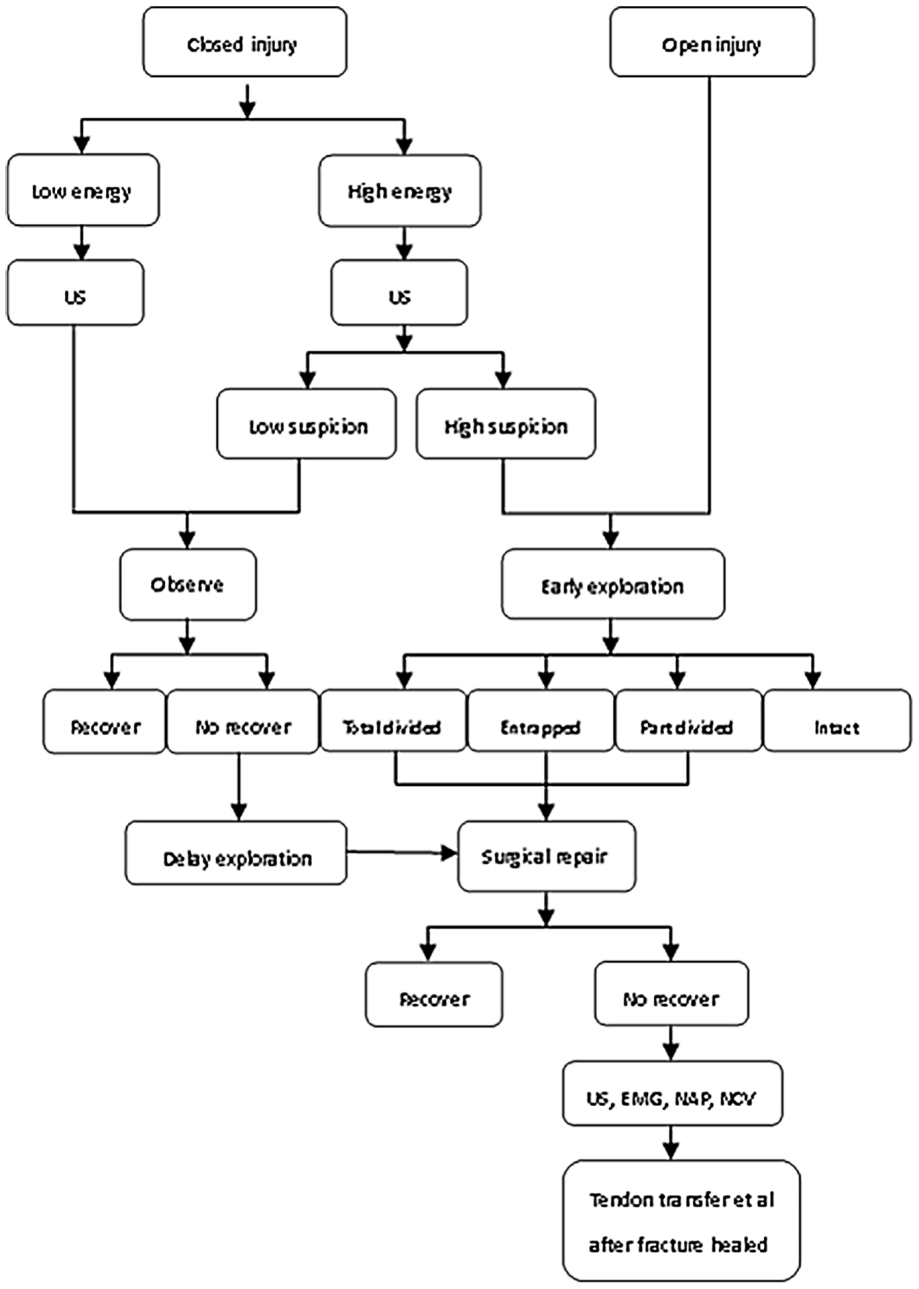
Management strategy of radial nerve injuries associated with humeral fractures (US: ultrasonography; EMG: electromyogram; NAP: nerve axonal physiology; NCV: nerve conduction velocity).

After comprehensively analyzing the above conclusions, we recommended that an early exploration was performed in all patients with open fractures. When patients suffer from a low energy closed injury, a delayed exploration should be performed if no nerve recovery was found with 5–8 weeks. If patients has a high energy closed injury, ultrasonography (US) should be used for evaluating the damage degree of radial nerve. If findings highly suggest a totally divided or entrapped injury, an early exploration should be performed. The exhaustive management strategy was shown in Fig. 4.

Although this study was regarded as a comprehensive systematic review of RS-based evidence for discussing the exploration time window of radial nerve injuries associated with humeral fractures, we acknowledged that this study has a number of limitations. The included studies were all retrospective studies with poor methodological quality. The general lack of random equence production and allocation concealment methods in the included RSs made it difficult to assess their methodological quality, thereby the risk of bias and potential to overestimate the effect may be existent. It is usually impossible to blind people accepting an operation, so performance bias is inevitable in the present meta-analysis. The different follow-up between trials was also considered to cause methodological heterogeneity. What’s more, we did not confine the language in the process of literature retrieval, but only German- and English-language trials were identified according to inclusion criteria, and this might result in language bias.

## Conclusion

The conservative strategy was a good choice for patients with low-energy closed fractures of humerus with radial nerve injury. We recommend early radial nerve exploration (within the first 2 weeks) in patients with open fractures or high-energy closed fractures of humerus with radial nerve injury. To draw a more convincing conclusion on the optimal management strategy, more methodologically improved trials with standardized outcome measures are recommended in future work.

## Supporting Information

Checklist S1 (DOC)
